# Supplementary material for: Navigating HPV vaccination challenges in low-resource settings: Practical strategies for estimating the size of out-of-school girls
Source: PLoS One. 2026 Oct 26;21(10):e0359273. doi: 10.1371/journal.pone.0359273 (PMC13704837; doi:10.1371/journal.pone.0359273)
Supplement: Supplementary file 1 [file pone.0359273.s001.docx]

**Key Informant Interview Guide**

**HPV Immunization Stakeholders**

**[*Notes:* START RECORDING]**

***Section I: Introduction***

*Let’s first talk about your role and the HPV vaccination programme in [COUNTRY NAME].*

1. **Tell me about your position and work with the HPV vaccination programme in your country.**
2. There are many different reasons why girls are out-of-school. **How are out-of-school girls segmented or distinguished by the immunization programme in your country? In other words, are there different subpopulations of out-of-school girls in your context?**
   a. How are these populations of out-of-school girls different or distinguishable from one another? (*Probe*: occupation, geography)
   b. What efforts have been made by the immunization programme in [*COUNTRY NAME*] to enumerate or quantify the population size(s) for out-of-school girls?
3. I would like to learn more about efforts to reach out-of-school girls with HPV vaccination in [*COUNTRY NAME*]. **What strategies have been implemented to increase HPV vaccination coverage among out-of-school girls?**
   a. Who is involved in implementing these strategies? (*Probe*: CSOs/NGOs, community health workers, facility-based health workers)
   b. For how long have these strategies been implemented?
   c. Where are these strategies implemented? Are they nationally implemented or geographically focused?

***Section II: Adoption/Planning/Pre-Implementation***

*I would like to learn more about the planning process for these strategies.*

4. **What did the planning phase for implementing these strategies look like?**
   a. Which stakeholders were mobilized and most critical to implementation planning?
   b. What resources and materials were required before formal implementation could begin? (*Probe*: financing, partnerships with religious leaders, training materials)
   c. Were there any obstacles or challenges during the planning phase that delayed implementation? If so, what were they, and how were they addressed?
5. **Is there anything you would have liked to see happen differently during the planning process? If so, what would that be?**

***Section III: Early/Mid/Late Implementation***

6. **Once plans were finalized, and these strategies were first implemented, to what extent did actual implementation deviate from the implementation plans?**
   a. What were some reasons that actual implementation deviated from what was initially planned?
7. **Since these strategies have been implemented, have there been any adaptations or changes to the strategies? If so, what changes have been made?**
   a. What was the reason these changes occurred?
   b. How long after initial implementation did these changes occur?
   c. Did these changes usher in any notable effects or impacts? If so, what were they?
8. **What have been some noteworthy successes of the strategies used to reach out-of-school girls with HPV vaccination?**
9. **What have been the biggest challenges of implementing these strategies to date?**
   a. What has been done to address some of these challenges?
   b. Is there anything else that should be done to address these implementation challenges?

***Section IV: Reach/Effectiveness***

10. **In your perspective, to what extent have these strategies been effective at increasing HPV vaccination coverage among out-of-school girls?**
    a. For which populations of out-of-school girls have these strategies increased HPV vaccination coverage?
    b. For which populations of out-of-school girls have these strategies been less successful? Why do you think that is?
    c. Are there groups or segments of the out-of-school population that are being missed or overlooked by these strategies? If so, who are they? What changes should be made to these strategies to reach them?
11. **Given the challenges increasing HPV vaccine coverage among out-of-school girls, what indicators or measures would you use to define a “successful” strategy for reaching out-of-school girls with HPV vaccination?**

***Section V: Maintenance/Sustainability***

*We’re at the end of our interview. Before we close, I wanted to ask you a few more questions.*

12. **What resources—whether material, financial, or human—are required to sustain implementation of these strategies to reach out-of-school girls?**
    a. Have you experienced any challenges accessing these resources in the past?
13. **Beyond the strategies we’ve discussed today, are there alternative strategies you think should be considered to better reach out-of-school girls?**
    a. What are these strategies?
    b. What might be some reasons these strategies haven’t been considered or implemented in [*COUNTRY NAME*]?
14. We are hoping to talk with other stakeholders from your country. **Are there other critical persons with whom you think we should speak to learn more about strategies for reaching out-of-school girls with HPV vaccination?**
    a. Is there anything you haven’t mentioned yet that you think is important for us to know about out-of-school girls or the HPV vaccination programme in your country?

**[*Notes:* END RECORDING]**

*Thank you very much for your time today. Please reach out to us if you have any questions about our research or if you think of other information that might be helpful for us to know.*
