# Supplementary material for: Navigating HPV vaccination challenges in low-resource settings: Practical strategies for estimating the size of out-of-school girls
Source: PLoS One. 2026 Oct 26;21(10):e0359273. doi: 10.1371/journal.pone.0359273 (PMC13704837; doi:10.1371/journal.pone.0359273)
Supplement: Supplementary file 2 [file pone.0359273.s003.docx]

PONE-D-26-19141

Thank you for considering the resubmission of our article entitled, “Navigating HPV Challenges in Low-Resource Settings: Practical Strategies for Estimating the Size of Out-of-School Girls.” We are appreciative of the thoughtful suggestions from the reviewers and have responded to each of the reviewers’ comments below. We believe that these changes improve the quality and clarity of this article.

We have marked the changes in the text, tables, and figures throughout. All changes in response to the reviewers have been highlighted in yellow in the main manuscript, and the page number and line numbers are indicated (included on the left side of the page).

Please let us know if you need any further information or clarification.

Thank you for your consideration of our manuscript.

Sincerely,

Soim Park

**Reviewer #1**

Overall, this is a well-conducted qualitative study exploring perspectives on estimating the number of out-of-school girls to enable adequate HPV vaccination coverage in low-resource settings. A major strength of the study is the recruitment of key informants from six countries. One limitation is the relatively small number of participants recruited from some of the countries, which fell below the initially planned sample size. However, this can be justified by the challenges associated with recruiting participants in these settings.
In general, the study is suitable for publication. However, I have a few minor comments:

*Response*: Thank you for the positive comments. We agree with the reviewer that the relatively small number of participants recruited from some countries is a limitation of this study. Recruitment depended on the availability and responsiveness of identified stakeholders, and despite up to four follow-up attempts, the number of participants in some countries remained below the initially planned sample size. Recruitment in Mozambique was particularly challenging due to the need to conduct interviews in Portuguese. We have added this information in limitation (page 25).

1. IRB clearance: The authors reported obtaining ethical approval from their home institution; however, it is unclear whether ethical approval or local permissions were also obtained from institutions in each of the countries from which participants were recruited.

*Response*: The study received an exemption determination from the Johns Hopkins Bloomberg School of Public Health Institutional Review Board (IRB). The study involved remote key informant interviews with stakeholders in their professional capacities and met the criteria for exemption because participants provided information based on their professional expertise rather than about personal experiences or opinions, without disclosing information that could place them at risk. The study was conducted solely by investigators at the US institution and did not involve local collaborating institutions or in-country data collection teams. Therefore, local ethics approvals were not sought in the participating countries.

2. The authors are advised to provide the interview guide used in the study as supplementary material.

*Response*: We have included the interview guide as a supplementary material.

3. In Figure 2, the top two rows are shaded too darkly, making the text difficult to read.

*Response*: In line with Reviewer 2’s comment #6, we have extensively revised Figure 2. These changes have been made on page 17.

**Reviewer #2**

This manuscript addresses an important public health challenge: identifying and reaching out-of-school (OOS) girls within HPV vaccination programs in low- and middle-income countries. The topic is highly relevant to achieving the WHO cervical cancer elimination targets and remains insufficiently explored in the existing literature. The qualitative design is appropriate for the research question, and the inclusion of stakeholders from multiple countries provides valuable insights into the practical realities of HPV vaccine implementation across diverse settings. Overall, the manuscript is well-written, clearly structured, and provides operational perspectives that may inform HPV vaccination program planning and implementation. The findings contribute to a better understanding of how OOS girls are defined, identified, and reached in different contexts. The following comments are offered for the authors’ consideration.

*Response*: We appreciate the reviewer’s thoughtful comments and believe that addressing them has strengthened the manuscript.

1. The manuscript describes several subgroups of OOS girls, including those affected by poverty, early marriage, pregnancy, disability, and displacement. However, HPV vaccination program in most participating countries primarily target girls aged 9–10 years, while school enrolment rates at the primary level appear relatively high. It would be helpful for the authors to clarify whether stakeholder discussions specifically referred to vaccine-eligible girls or to OOS girls more broadly. In addition, the authors may wish to discuss the relevance of these findings in the context of catch-up HPV vaccination programs, where older adolescent girls may constitute an important target population and the identified typologies may be particularly applicable.

Response: We thank the reviewer for requesting this clarification. At the beginning of each interview, we explicitly framed the discussion around OOS girls eligible for HPV vaccination within the target age range of each country’s program, rather than OOS girls more broadly. We have clarified in line 96 on page 7 that Cambodia targeted girls aged 9-14 years, while the other countries targeted girls aged 9- or 10-years. We have also clarified on page 8 that we recruited participants with experiences on reaching OOS girls eligible for HPV vaccination and that our study focused specifically on this population rather than OOS girls in general. On page 11, we have specified that our focus is the OOS girls within the HPV vaccination target age range.

Furthermore, we have added on page 22 (lines 428-430), that typologies can be used to inform catch-up vaccination programs.

2. Additional details regarding the qualitative analysis process would strengthen the methodological transparency of the study. While the manuscript notes that three researchers participated in coding and the refinement of the coding framework, it is unclear whether interviews were coded independently, how coding discrepancies were resolved, whether any assessment of coding consistency was undertaken, and what proportion of transcripts were reviewed by multiple coders.

*Response*: We have added further details regarding the qualitative analysis process on pages 10-11. All three coders had graduate-level training in qualitative research and 5-15 years of qualitative research experiences. Each transcript was independently coded by one of the three coders; transcripts were not routinely double-coded. Rather than double-coding a subset of transcripts or conducting a formal assessment of intercoder reliability, the three coders met weekly throughout the analysis to review the application of codes, discuss emerging themes, refine code definitions as needed, and resolve questions or discrepancies in coding through consensus. Thus, coding consistency was maintained through ongoing team-based review and discussion rather than through quantitative assessment of intercoder agreement.

3. The proposed typology of OOS girls represents one of the central findings of the study. However, the process by which this typology was developed could be described in greater detail. It would be helpful to clarify whether the categories emerged entirely inductively from the data or were informed by pre-existing conceptual frameworks, and the extent to which they were represented across interviews.

Response: OOS typology was developed inductively, rather than using the pre-existing conceptual frameworks that are largely developed by the field of education. Because we thought OOS typology in public health may be different from that developed by another field, we did not confine ourselves into a priori category. We have highlighted this on page 10, that data analysis was largely inductive.

4. The manuscript would benefit from a brief description of how target populations for HPV vaccination are typically estimated during routine programs planning in LMICs. Providing this context would help readers better understand why enumeration of OOS girls presents a unique challenge and how the approaches described in this study fit within existing vaccination planning frameworks.

*Response*: We thank the reviewer for this suggestion. We have added a description of typical enumeration approaches for routine childhood vaccination, how enumeration for HPV vaccination differs, and the specific challenges associated with enumerating HPV vaccine-eligible girls (in lines 61-71 on page 6).

Added citations:

- World Health Organization. Immunization in Practice: A practical guide for health staff 2015 update. Geneva, Switzerland: World Health Organization; 2015.
- Mafigiri DK, Iradukunda C, Atumanya C, Odie M, Mancuso A, Tran N, McGrath J, Luzze H. A qualitative study of the development and utilization of health facility-based immunization microplans in Uganda. Health Res Policy Syst 2021 Aug 11;19(Suppl 2):52. PMID:34380523

5. The authors may consider expanding the recommendations section. Given the comprehensiveness of the findings, the manuscript would benefit from more detailed and actionable guidance regarding the identification and enumeration of OOS girls.

*Response*: We have expanded the recommendations section. The core recommendations are the same, but we have reorganized them based on the planning, implementation, and evaluation stages and added further detail to each.

6. The authors may consider refining Figure 2 to provide more detail on the proposed framework and improve its accessibility and interpretation.

*Response*: We have extensively revised Figure 2 to improve interpretability. These are reflected on page 17.

7. The manuscript would benefit from providing the semi-structured interview guide as supplementary material. This would improve transparency and help readers better understand how key themes were explored during the interviews.

*Response*: In line with Reviewer 1’s comment #2, we have included the interview guide as a supplementary material.

In summary, this study addresses an important implementation challenge in HPV vaccination programs and contributes valuable insights regarding the identification and enumeration of OOS girls. The comments above are intended to further strengthen the methodological transparency, interpretation, and practical applicability of the findings.
