# Supplementary material for: Navigating HPV vaccination challenges in low-resource settings: Practical strategies for estimating the size of out-of-school girls
Source: PLoS One. 2026 Oct 26;21(10):e0359273. doi: 10.1371/journal.pone.0359273 (PMC13704837; doi:10.1371/journal.pone.0359273)
Supplement: Peer review history

# Peer review: Decision Letter 0

**DOI:** 10.1371/journal.pone.0359273.r001

**Authors:** Gulzhanat Aimagambetova

12 Jun 2026

Dear Dr. Park,

plosone@plos.org. When you're ready to submit your revision, log on to https://www.editorialmanager.com/pone/ and select the 'Submissions Needing Revision' folder to locate your manuscript file.

- A letter that responds to each point raised by the academic editor and reviewer(s). You should upload this letter as a separate file labeled 'Response to Reviewers'.
- A marked-up copy of your manuscript that highlights changes made to the original version. You should upload this as a separate file labeled 'Revised Manuscript with Track Changes'.
- An unmarked version of your revised paper without tracked changes. You should upload this as a separate file labeled 'Manuscript'.

If you would like to make changes to your financial disclosure, please include your updated statement in your cover letter. Guidelines for resubmitting your figure files are available below the reviewer comments at the end of this letter.

If applicable, we recommend that you deposit your laboratory protocols in protocols.io to enhance the reproducibility of your results. Protocols.io assigns your protocol its own identifier (DOI) so that it can be cited independently in the future. For instructions see: https://journals.plos.org/plosone/s/submission-guidelines#loc-laboratory-protocols. Additionally, PLOS ONE offers an option for publishing peer-reviewed Lab Protocol articles, which describe protocols hosted on protocols.io. Read more information on sharing protocols at https://plos.org/protocols?utm_medium=editorial-email&utm_source=authorletters&utm_campaign=protocols.

As the corresponding author, your ORCID iD is verified in the submission system and will appear in the published article. PLOS supports the use of ORCID, and we encourage all coauthors to register for an ORCID iD and use it as well. Please encourage your coauthors to verify their ORCID iD within the submission system before final acceptance, as unverified ORCID iDs will not appear in the published article. *Only*  the individual author can complete the verification step; PLOS staff *cannot*  verify ORCID iDs on behalf of authors.

We look forward to receiving your revised manuscript.

Kind regards,

Gulzhanat Aimagambetova

Academic Editor

PLOS One

**Journal Requirements:**

When submitting your revision, we need you to address these additional requirements.

1. Please ensure that your manuscript meets PLOS ONE’s style requirements, including those for file naming. The PLOS ONE style templates can be found at

https://journals.plos.org/plosone/s/file?id=wjVg/PLOSOne_formatting_sample_main_body.pdf and

https://journals.plos.org/plosone/s/file?id=ba62/PLOSOne_formatting_sample_title_authors_affiliations.pdf

2. We note that the grant information you provided in the ‘Funding Information’ and ‘Financial Disclosure’ sections do not match.

When you resubmit, please ensure that you provide the correct grant numbers for the awards you received for your study in the ‘Funding Information’ section.

3. Thank you for stating the following financial disclosure:

“This study was funded by the HAPPI Consortium via the Gates Foundation (INV-046461, INV-057603). JGR acknowledges support from the National Institute of Mental Health (R25MH083620).”

Please state what role the funders took in the study.  If the funders had no role, please state: "The funders had no role in study design, data collection and analysis, decision to publish, or preparation of the manuscript."

If this statement is not correct you must amend it as needed.

Please include this amended Role of Funder statement in your cover letter; we will change the online submission form on your behalf.

4. Please note that funding information should not appear in any section or other areas of your manuscript. We will only publish funding information present in the Funding Statement section of the online submission form. Please remove any funding-related text from the manuscript.

5. In the online submission form, you indicated that [ Data are not publicly available but are available upon reasonable request ].

All PLOS journals now require all data underlying the findings described in their manuscript to be freely available to other researchers, either 1. In a public repository, 2. Within the manuscript itself, or 3. Uploaded as supplementary information.

This policy applies to all data except where public deposition would breach compliance with the protocol approved by your research ethics board. If your data cannot be made publicly available for ethical or legal reasons (e.g., public availability would compromise patient privacy), please explain your reasons on resubmission and your exemption request will be escalated for approval.

6. Please include your full ethics statement in the ‘Methods’ section of your manuscript file. In your statement, please include the full name of the IRB or ethics committee who approved or waived your study, as well as whether or not you obtained informed written or verbal consent. If consent was waived for your study, please include this information in your statement as well.

7. Please remove your figures from within your manuscript file, leaving only the individual TIFF/EPS image files, uploaded separately. These will be automatically included in the reviewers’ PDF.

8. If the reviewer comments include a recommendation to cite specific previously published works, please review and evaluate these publications to determine whether they are relevant and should be cited. There is no requirement to cite these works unless the editor has indicated otherwise.

9. Please review your reference list to ensure that it is complete and correct. If you have cited papers that have been retracted, please include the rationale for doing so in the manuscript text, or remove these references and replace them with relevant current references. Any changes to the reference list should be mentioned in the rebuttal letter that accompanies your revised manuscript. If you need to cite a retracted article, indicate the article’s retracted status in the References list and also include a citation and full reference for the retraction notice.

[Note: HTML markup is below. Please do not edit.]

Reviewers' comments:

Reviewer’s Responses to Questions

**Comments to the Author**

1. Is the manuscript technically sound, and do the data support the conclusions?

Reviewer #1: Yes

Reviewer #2: Yes

**********

2. Has the statistical analysis been performed appropriately and rigorously?

Reviewer #1: N/A

Reviewer #2: N/A

**********

3. Have the authors made all data underlying the findings in their manuscript fully available?

The PLOS Data policy

Reviewer #1: No

Reviewer #2: Yes

**********

4. Is the manuscript presented in an intelligible fashion and written in standard English?

Reviewer #1: Yes

Reviewer #2: Yes

**********

Reviewer #1: Overall, this is a well-conducted qualitative study exploring perspectives on estimating the number of out-of-school girls to enable adequate HPV vaccination coverage in low-resource settings. A major strength of the study is the recruitment of key informants from six countries. One limitation is the relatively small number of participants recruited from some of the countries, which fell below the initially planned sample size. However, this can be justified by the challenges associated with recruiting participants in these settings.

In general, the study is suitable for publication. However, I have a few minor comments:

1. IRB clearance: The authors reported obtaining ethical approval from their home institution; however, it is unclear whether ethical approval or local permissions were also obtained from institutions in each of the countries from which participants were recruited.

2. The authors are advised to provide the interview guide used in the study as supplementary material.

3. In Figure 2, the top two rows are shaded too darkly, making the text difficult to read.

Reviewer #2: This manuscript addresses an important public health challenge: identifying and reaching out-of-school (OOS) girls within HPV vaccination programs in low- and middle-income countries. The topic is highly relevant to achieving the WHO cervical cancer elimination targets and remains insufficiently explored in the existing literature. The qualitative design is appropriate for the research question, and the inclusion of stakeholders from multiple countries provides valuable insights into the practical realities of HPV vaccine implementation across diverse settings. Overall, the manuscript is well-written, clearly structured, and provides operational perspectives that may inform HPV vaccination program planning and implementation. The findings contribute to a better understanding of how OOS girls are defined, identified, and reached in different contexts. The following comments are offered for the authors’ consideration.

1. The manuscript describes several subgroups of OOS girls, including those affected by poverty, early marriage, pregnancy, disability, and displacement. However, HPV vaccination program in most participating countries primarily target girls aged 9–10 years, while school enrolment rates at the primary level appear relatively high. It would be helpful for the authors to clarify whether stakeholder discussions specifically referred to vaccine-eligible girls or to OOS girls more broadly. In addition, the authors may wish to discuss the relevance of these findings in the context of catch-up HPV vaccination programs, where older adolescent girls may constitute an important target population and the identified typologies may be particularly applicable.

2. Additional details regarding the qualitative analysis process would strengthen the methodological transparency of the study. While the manuscript notes that three researchers participated in coding and the refinement of the coding framework, it is unclear whether interviews were coded independently, how coding discrepancies were resolved, whether any assessment of coding consistency was undertaken, and what proportion of transcripts were reviewed by multiple coders.

3. The proposed typology of OOS girls represents one of the central findings of the study. However, the process by which this typology was developed could be described in greater detail. It would be helpful to clarify whether the categories emerged entirely inductively from the data or were informed by pre-existing conceptual frameworks, and the extent to which they were represented across interviews.

4. The manuscript would benefit from a brief description of how target populations for HPV vaccination are typically estimated during routine programs planning in LMICs. Providing this context would help readers better understand why enumeration of OOS girls presents a unique challenge and how the approaches described in this study fit within existing vaccination planning frameworks.

5. The authors may consider expanding the recommendations section. Given the comprehensiveness of the findings, the manuscript would benefit from more detailed and actionable guidance regarding the identification and enumeration of OOS girls.

6. The authors may consider refining Figure 2 to provide more detail on the proposed framework and improve its accessibility and interpretation.

7. The manuscript would benefit from providing the semi-structured interview guide as supplementary material. This would improve transparency and help readers better understand how key themes were explored during the interviews.

In summary, this study addresses an important implementation challenge in HPV vaccination programs and contributes valuable insights regarding the identification and enumeration of OOS girls. The comments above are intended to further strengthen the methodological transparency, interpretation, and practical applicability of the findings.

**********

what does this mean?). If published, this will include your full peer review and any attached files.

If you choose “no”, your identity will remain anonymous but your review may still be made public.

**Do you want your identity to be public for this peer review?**  For information about this choice, including consent withdrawal, please see our Privacy Policy

Reviewer #1: No

Reviewer #2: **Yes:**  Fatima Kassymbekova, Asfendiyarov Kazakh National Medical University

**********

[NOTE: If reviewer comments were submitted as an attachment file, they will be attached to this email and accessible via the submission site. Please log into your account, locate the manuscript record, and check for the action link "View Attachments". If this link does not appear, there are no attachment files.]

To ensure your figures meet our technical requirements, please review our figure guidelines: https://journals.plos.org/plosone/s/figures

You may also use PLOS’s free figure tool, NAAS, to help you prepare publication quality figures: https://journals.plos.org/plosone/s/figures#loc-tools-for-figure-preparation.

NAAS will assess whether your figures meet our technical requirements by comparing each figure against our figure specifications.

# Author comment: Author response to Decision Letter 1

**DOI:** 10.1371/journal.pone.0359273.r002

8 Sep 2026

PONE-D-26-19141

Thank you for considering the resubmission of our article entitled, “Navigating HPV Challenges in Low-Resource Settings: Practical Strategies for Estimating the Size of Out-of-School Girls.” We are appreciative of the thoughtful suggestions from the reviewers and have responded to each of the reviewers’ comments below. We believe that these changes improve the quality and clarity of this article.

We have marked the changes in the text, tables, and figures throughout. All changes in response to the reviewers have been highlighted in yellow in the main manuscript, and the page number and line numbers are indicated (included on the left side of the page).

Please let us know if you need any further information or clarification.

Thank you for your consideration of our manuscript.

Sincerely,

Soim Park

Reviewer #1

Overall, this is a well-conducted qualitative study exploring perspectives on estimating the number of out-of-school girls to enable adequate HPV vaccination coverage in low-resource settings. A major strength of the study is the recruitment of key informants from six countries. One limitation is the relatively small number of participants recruited from some of the countries, which fell below the initially planned sample size. However, this can be justified by the challenges associated with recruiting participants in these settings.

In general, the study is suitable for publication. However, I have a few minor comments:

Response: Thank you for the positive comments. We agree with the reviewer that the relatively small number of participants recruited from some countries is a limitation of this study. Recruitment depended on the availability and responsiveness of identified stakeholders, and despite up to four follow-up attempts, the number of participants in some countries remained below the initially planned sample size. Recruitment in Mozambique was particularly challenging due to the need to conduct interviews in Portuguese. We have added this information in limitation (page 25).

1. IRB clearance: The authors reported obtaining ethical approval from their home institution; however, it is unclear whether ethical approval or local permissions were also obtained from institutions in each of the countries from which participants were recruited.

Response: The study received an exemption determination from the Johns Hopkins Bloomberg School of Public Health Institutional Review Board (IRB). The study involved remote key informant interviews with stakeholders in their professional capacities and met the criteria for exemption because participants provided information based on their professional expertise rather than about personal experiences or opinions, without disclosing information that could place them at risk. The study was conducted solely by investigators at the US institution and did not involve local collaborating institutions or in-country data collection teams. Therefore, local ethics approvals were not sought in the participating countries.

2. The authors are advised to provide the interview guide used in the study as supplementary material.

Response: We have included the interview guide as a supplementary material.

3. In Figure 2, the top two rows are shaded too darkly, making the text difficult to read.

Response: In line with Reviewer 2’s comment #6, we have extensively revised Figure 2. These changes have been made on page 17.

Reviewer #2

This manuscript addresses an important public health challenge: identifying and reaching out-of-school (OOS) girls within HPV vaccination programs in low- and middle-income countries. The topic is highly relevant to achieving the WHO cervical cancer elimination targets and remains insufficiently explored in the existing literature. The qualitative design is appropriate for the research question, and the inclusion of stakeholders from multiple countries provides valuable insights into the practical realities of HPV vaccine implementation across diverse settings. Overall, the manuscript is well-written, clearly structured, and provides operational perspectives that may inform HPV vaccination program planning and implementation. The findings contribute to a better understanding of how OOS girls are defined, identified, and reached in different contexts. The following comments are offered for the authors’ consideration.

Response: We appreciate the reviewer’s thoughtful comments and believe that addressing them has strengthened the manuscript.

1. The manuscript describes several subgroups of OOS girls, including those affected by poverty, early marriage, pregnancy, disability, and displacement. However, HPV vaccination program in most participating countries primarily target girls aged 9–10 years, while school enrolment rates at the primary level appear relatively high. It would be helpful for the authors to clarify whether stakeholder discussions specifically referred to vaccine-eligible girls or to OOS girls more broadly. In addition, the authors may wish to discuss the relevance of these findings in the context of catch-up HPV vaccination programs, where older adolescent girls may constitute an important target population and the identified typologies may be particularly applicable.

Response: We thank the reviewer for requesting this clarification. At the beginning of each interview, we explicitly framed the discussion around OOS girls eligible for HPV vaccination within the target age range of each country’s program, rather than OOS girls more broadly. We have clarified in line 96 on page 7 that Cambodia targeted girls aged 9-14 years, while the other countries targeted girls aged 9- or 10-years. We have also clarified on page 8 that we recruited participants with experiences on reaching OOS girls eligible for HPV vaccination and that our study focused specifically on this population rather than OOS girls in general. On page 11, we have specified that our focus is the OOS girls within the HPV vaccination target age range.

Furthermore, we have added on page 22 (lines 428-430), that typologies can be used to inform catch-up vaccination programs.

2. Additional details regarding the qualitative analysis process would strengthen the methodological transparency of the study. While the manuscript notes that three researchers participated in coding and the refinement of the coding framework, it is unclear whether interviews were coded independently, how coding discrepancies were resolved, whether any assessment of coding consistency was undertaken, and what proportion of transcripts were reviewed by multiple coders.

Response: We have added further details regarding the qualitative analysis process on pages 10-11. All three coders had graduate-level training in qualitative research and 5-15 years of qualitative research experiences. Each transcript was independently coded by one of the three coders; transcripts were not routinely double-coded. Rather than double-coding a subset of transcripts or conducting a formal assessment of intercoder reliability, the three coders met weekly throughout the analysis to review the application of codes, discuss emerging themes, refine code definitions as needed, and resolve questions or discrepancies in coding through consensus. Thus, coding consistency was maintained through ongoing team-based review and discussion rather than through quantitative assessment of intercoder agreement.

3. The proposed typology of OOS girls represents one of the central findings of the study. However, the process by which this typology was developed could be described in greater detail. It would be helpful to clarify whether the categories emerged entirely inductively from the data or were informed by pre-existing conceptual frameworks, and the extent to which they were represented across interviews.

Response: OOS typology was developed inductively, rather than using the pre-existing conceptual frameworks that are largely developed by the field of education. Because we thought OOS typology in public health may be different from that developed by another field, we did not confine ourselves into a priori category. We have highlighted this on page 10, that data analysis was largely inductive.

4. The manuscript would benefit from a brief description of how target populations for HPV vaccination are typically estimated during routine programs planning in LMICs. Providing this context would help readers better understand why enumeration of OOS girls presents a unique challenge and how the approaches described in this study fit within existing vaccination planning frameworks.

Response: We thank the reviewer for this suggestion. We have added a description of typical enumeration approaches for routine childhood vaccination, how enumeration for HPV vaccination differs, and the specific challenges associated with enumerating HPV vaccine-eligible girls (in lines 61-71 on page 6).

Added citations:

• World Health Organization. Immunization in Practice: A practical guide for health staff 2015 update. Geneva, Switzerland: World Health Organization; 2015.

• Mafigiri DK, Iradukunda C, Atumanya C, Odie M, Mancuso A, Tran N, McGrath J, Luzze H. A qualitative study of the development and utilization of health facility-based immunization microplans in Uganda. Health Res Policy Syst 2021 Aug 11;19(Suppl 2):52. PMID:34380523

5. The authors may consider expanding the recommendations section. Given the comprehensiveness of the findings, the manuscript would benefit from more detailed and actionable guidance regarding the identification and enumeration of OOS girls.

Response: We have expanded the recommendations section. The core recommendations are the same, but we have reorganized them based on the planning, implementation, and evaluation stages and added further detail to each.

6. The authors may consider refining Figure 2 to provide more detail on the proposed framework and improve its accessibility and interpretation.

Response: We have extensively revised Figure 2 to improve interpretability. These are reflected on page 17.

7. The manuscript would benefit from providing the semi-structured interview guide as supplementary material. This would improve transparency and help readers better understand how key themes were explored during the interviews.

Response: In line with Reviewer 1’s comment #2, we have included the interview guide as a supplementary material.

In summary, this study addresses an important implementation challenge in HPV vaccination programs and contributes valuable insights regarding the identification and enumeration of OOS girls. The comments above are intended to further strengthen the methodological transparency, interpretation, and practical applicability of the findings.

Attachment Submitted filename: PLOS One Reply letter.docx

# Peer review: Decision Letter 1

**DOI:** 10.1371/journal.pone.0359273.r003

**Authors:** Gulzhanat Aimagambetova

13 Sep 2026

Navigating HPV Vaccination Challenges in Low-Resource Settings: Practical Strategies for Estimating the Size of Out-of-School Girls

PONE-D-26-19141R1

Dear Dr. Soim Park,

We’re pleased to inform you that your manuscript has been judged scientifically suitable for publication and will be formally accepted for publication once it meets all outstanding technical requirements.

Please make sure to clearly state the ethical approval in the manuscript, including the approving board, date, and protocol number.

Within one week, you’ll receive an e-mail detailing the required amendments. When these have been addressed, you’ll receive a formal acceptance letter and your manuscript will be scheduled for publication.

An invoice will be generated when your article is formally accepted. Please note, if your institution has a publishing partnership with PLOS and your article meets the relevant criteria, all or part of your publication costs will be covered. Please make sure your user information is up-to-date by logging into Editorial Manager at Editorial Manager® and clicking the ‘Update My Information' link at the top of the page. For questions related to billing, please contact billing support.

If your institution or institutions have a press office, please notify them about your upcoming paper to help maximize its impact. If they’ll be preparing press materials, please inform our press team as soon as possible -- no later than 48 hours after receiving the formal acceptance. Your manuscript will remain under strict press embargo until 2 pm Eastern Time on the date of publication. For more information, please contact onepress@plos.org.

Kind regards,

Gulzhanat Aimagambetova

Academic Editor

PLOS One

Additional Editor Comments (optional):

Reviewers' comments:

Reviewer’s Responses to Questions

**Comments to the Author**

Reviewer #1: All comments have been addressed

Reviewer #2: All comments have been addressed

**********

2. Is the manuscript technically sound, and do the data support the conclusions?

Reviewer #1: Yes

Reviewer #2: (No Response)

**********

3. Has the statistical analysis been performed appropriately and rigorously?

Reviewer #1: N/A

Reviewer #2: (No Response)

**********

4. Have the authors made all data underlying the findings in their manuscript fully available?

The PLOS Data policy

Reviewer #1: No

Reviewer #2: (No Response)

**********

5. Is the manuscript presented in an intelligible fashion and written in standard English?

Reviewer #1: Yes

Reviewer #2: (No Response)

**********

Reviewer #1: (No Response)

Reviewer #2: (No Response)

**********

what does this mean?). If published, this will include your full peer review and any attached files.

If you choose “no”, your identity will remain anonymous but your review may still be made public.

**Do you want your identity to be public for this peer review?**  For information about this choice, including consent withdrawal, please see our Privacy Policy

Reviewer #1: No

Reviewer #2: No

**********

# Editor report: Acceptance letter

**DOI:** 10.1371/journal.pone.0359273.r004

**Authors:** Gulzhanat Aimagambetova

PONE-D-26-19141R1

PLOS One

Dear Dr. Park,

I'm pleased to inform you that your manuscript has been deemed suitable for publication in PLOS One. Congratulations! Your manuscript is now being handed over to our production team.

At this stage, our production department will prepare your paper for publication. This includes ensuring the following:

* All references, tables, and figures are properly cited

* All relevant supporting information is included in the manuscript submission,

* There are no issues that prevent the paper from being properly typeset

You will receive further instructions from the production team, including instructions on how to review your proof when it is ready. Please keep in mind that we are working through a large volume of accepted articles, so please give us a few days to review your paper and let you know the next and final steps.

Lastly, if your institution or institutions have a press office, please let them know about your upcoming paper now to help maximize its impact. If they'll be preparing press materials, please inform our press team within the next 48 hours. Your manuscript will remain under strict press embargo until 2 pm Eastern Time on the date of publication. For more information, please contact onepress@plos.org.

You will receive an invoice from PLOS for your publication fee after your manuscript has reached the completed accept phase. If you receive an email requesting payment before acceptance or for any other service, this may be a phishing scheme. Learn how to identify phishing emails and protect your accounts at https://explore.plos.org/phishing.

If we can help with anything else, please email us at customercare@plos.org.

Thank you for submitting your work to PLOS One and supporting open access.

Kind regards,

PLOS One Editorial Office Staff

on behalf of

Dr. Gulzhanat Aimagambetova

Academic Editor

PLOS One
